# Healthcare providers’ perceptions of barriers and facilitators to implementation of an acute traumatic brain injury clinical practice guideline: a CFIR-informed qualitative study at a regional referral hospital in Northern Tanzania

**DOI:** 10.64898/2026.10.04.26364725

**Published:** 2026-10-06

**Authors:** Vivian F. Saria, Gregory G. Zaccheus, Joao Ricardo Nickenig Vissoci, Francis Sakita, Winfrida C. Mwita, Linda Minja, Timothy Antipas Peter, João Vítor Perez de Souza, Kim Madundo, Blandina T. Mmbaga, Catherine A. Staton, Anna Tupetz

## Abstract

**Background:**

Traumatic brain injury (TBI) is a major cause of morbidity and mortality in low-and middle-income countries (LMICs), where the burden of injury is disproportionately high, and healthcare resources are often limited. Clinical Practice Guidelines (CPGs) improve the consistency and quality of TBI care by shifting medical decision-making from individual judgement to evidence-based recommendations. The strong link between improved TBI outcomes and CPG adoption has largely been established in high-income country (HIC) hospital settings; evidence on implementing TBI CPGs in LMIC hospital settings is limited.

**Objective:**

This study explored healthcare providers’ experiences to identify barriers and facilitators to implementing a TBI CPG in the Emergency Department (EMD) at Kilimanjaro Christian Medical Center in Northern Tanzania, guided by the Consolidated Framework for Implementation Research 2.0 (CFIR).

**Methods:**

We conducted semi-structured in-depth interviews (IDIs) with doctors and nurses who regularly managed TBI patients at the EMD. We purposively sampled participants between June and August 2023. The research team analysed data using thematic content analysis guided by CFIR domains, developing codes and memos iteratively.

**Results:**

7 nurses and 8 doctors with an average of 5 years of experience caring for patients in the EMD completed in-person, in-depth interviews. Most providers reported frequent exposure to TBI cases and regular use of the CPG in routine clinical practice. However, not all participants reported consistent CPG use, highlighting variation in implementation among providers. Key facilitators included strong interprofessional teamwork, readily accessible guideline materials in clinical areas, availability of diagnostic imaging, and perceived improvements in patient outcomes. Barriers to consistent adherence included ICU bed shortages, limited operating theatre capacity, equipment and medication stock-outs, high patient volumes, staffing pressures, and a lack of structured refresher training or formal audit and feedback mechanisms. Variability in procedural confidence, particularly among less experienced providers, also affected implementation consistency.

**Conclusion:**

Our findings show that a TBI CPG is well accepted and largely integrated into routine care among experienced providers. However, sustainability depends on strengthening training, mentorship, audit, and feedback systems for CPG use and on addressing system-level resource constraints. Institutional investment in capacity building is essential to ensure consistent and sustained CPG uptake in our setting.

## Full Text Availability

The license terms selected by the author(s) for this preprint version do not permit archiving in PMC. The full text is available from the preprint server.

